# Assessment of acoustic pulse therapy (APT), a non-antibiotic treatment for dairy cows with clinical and subclinical mastitis

**DOI:** 10.1371/journal.pone.0199195

**Published:** 2018-07-10

**Authors:** Gabriel Leitner, David Zilberman, Eduard Papirov, Sela Shefy

**Affiliations:** 1 National Mastitis Reference Center, Kimron Veterinary Institute, Bet Dagan, Israel; 2 Department of Agricultural and Resource Economics, University of California, Berkeley, United States of America; 3 HI-Impacts, Raanana, Israel; University of Illinois, UNITED STATES

## Abstract

Clinical and subclinical mastitis affects 30% of cows and is regarded as the most significant economic burden on the dairy farm reducing milk yield and quality and increasing culling rate. A proprietary Acoustic Pulse Therapy (APT) device was developed specifically for treating dairy cows. The APT device was designed to produce deep penetrating acoustic pulses that are distributed over a large treated area at a therapeutic level. This paper presents findings from a clinical assessment of this technology for the treatment of dairy cows with subclinical and clinical mastitis. In subclinical mastitis, a group of 116 cows from 3 herds were identified with subclinical intramammary infection and enrolled in the study; 78 cows were assigned to the treatment group and 38 cows to the control group. Significant differences (*P*<0.001) were found where 70.5% of the cows in the treatment group returned to normal milk production, compared with only 18.4% of the control group. Daily milk yields of the treated cows increased significantly (*P<0*.*05*) and the percentage of cows with log somatic cell count under 5.6 cells/mL was significantly higher (*P<0*.*001*). Milk of the infected quarters appeared normal with lactose greater than 4.8%, but this difference was not significant. Of the treated cows with identified bacteria, 52.6% of the quarters were cured, while in the control group only 25.0% (*P<0*.*001*). Specifically, all cows identified with *Escherichia coli* in the treatment group were cured, with 66.6% cured with no intervention in the control. Spontaneous cure of glands infected with coagulase negative staphylococci (CNS) and *Streptococci* was low while treatment successfully increased the cure of CNS from 13.3% to 53.8% and that of *Streptococci* from 18.2% to 36.4%. Of the 4 cows identified with *Staphylococcus aureus*, 3 were cured. The clinical mastitis study group included 29 infected cows that were submitted either to a gold standard antibiotic treatment subgroup of 16 cows (n = 16) or to an APT treatment subgroup of 13 cows (n = 13). A cure of 18.7% was shown for the antibiotic treatment, of which logSCC returned to <5.6 cell/mL and 56.2% were culled. A cure of 76.9% was shown for the APT treatment with only one cow culled (7.7%).

## Introduction

Due to economic pressure dairy farmers exert continuous efforts to maximize profitability by constant improvement of genetic selection, nutrition, and herd management. Thus, an increase in herd size, to include thousands of cows, led to the development of a concept that cow herd management and health control should be primarily focused on the herd rather than on the individual cow [1]. Computerized data acquisition, which provides on-line information on cows’ milk yield, milk composition and animal health, and opens new options to focus on the individual cow [2].

Mastitis is the single most important factor that imposes economic burdens on dairy farms worldwide [3]. It is estimated that mastitis infections affect 30% of dairy cattle and cost the EU dairy industry about €1.55 billion in 2005 [4] and the US industry US $2 billion [5]. Mastitis is normally divided into clinical and subclinical infection, both result in decreased milk yield, deterioration in milk quality and increased risk of culling [6,7]. The major causes of mastitis are bacterial: coliforms, *Streptococci*, coagulase-positive staphylococci (mainly *Staphylococcus aureus*) and coagulase-negative staphylococci (CNS). Cows are most susceptible to bacterial infection after drying-off and prior to calving, with symptoms becoming apparent in early lactation [8].

It is obligatory to treat clinical mastitis due to animal welfare standards. Thus, control of clinical mastitis during lactation includes administration of antibiotics or non-steroidal anti-inflammatory drugs (NSAID)[9]. Therefore, it is compulsory to segregate milk obtained from the infected quarter during treatment until full recovery of the gland, or to obliterate the infected gland or cull the cow. However, antibiotic treatment of mastitis is not completely effective against environmental pathogens [10]. Moreover, during treatment, milk is discarded due to the presence of abnormal milk and antibiotic residues which tremendously increase the economic losses.

Treatment of subclinical mastitis presents different challenges due to its wide prevalence that could reach 20 to 40% of the udders in some herds [4, 11]. Due to the difficulty in identification of the subclinical stage, many of the cows with subclinical chronic infection are not noticed because there are no recognizable symptoms and the milk appears visually normal.

Cows have four independent milk producing mammary glands, where each gland is independent of the others and is also referred to as a ‘quarter’. In most cases of intramammary infection (IMI), only one gland is involved. Thus, since somatic cell count (SCC) in the milk increases exponentially, its number is notable on the cow level, as well as its milk yield and quality. Therefore, the cow is profitable and considered healthy and milk quality is considered suitable for human consumption, despite frequent, unnoted cases of subclinical IMI of the animal. Nevertheless, on the gland level, the presence of the bacteria lead to milk containing high SCC and other changes in its composition related to the infection, such as decrease in lactose level [7] and changes in protein composition [12,13], which are generally disregarded.

Routine monthly milk recording, including SCC, is a practical procedure in many countries, which could serve as a basis for determining the presence of IMI and relevant treatment decisions [14,15]. However, the elapsed time between two consecutive tests is not sufficient for identifying the necessary warning signs or for performing the analysis necessary for decision making. Therefore, it is not simple to decide whether to treat or to ignore IMI in cows.

In the case of subclinical mastitis, costs of medicine and milk loss due to antibiotic treatment of cows that are not at risk needs to be justified [14–16]. Moreover, because antibiotic treatment costs during lactation are high and there is significant concern of overuse of drugs such as antibiotics, which may increase the number of humans and animals infected by antibiotic-resistant bacteria [17], most animals are treated only during the dry-off period.

**Fig 1:**
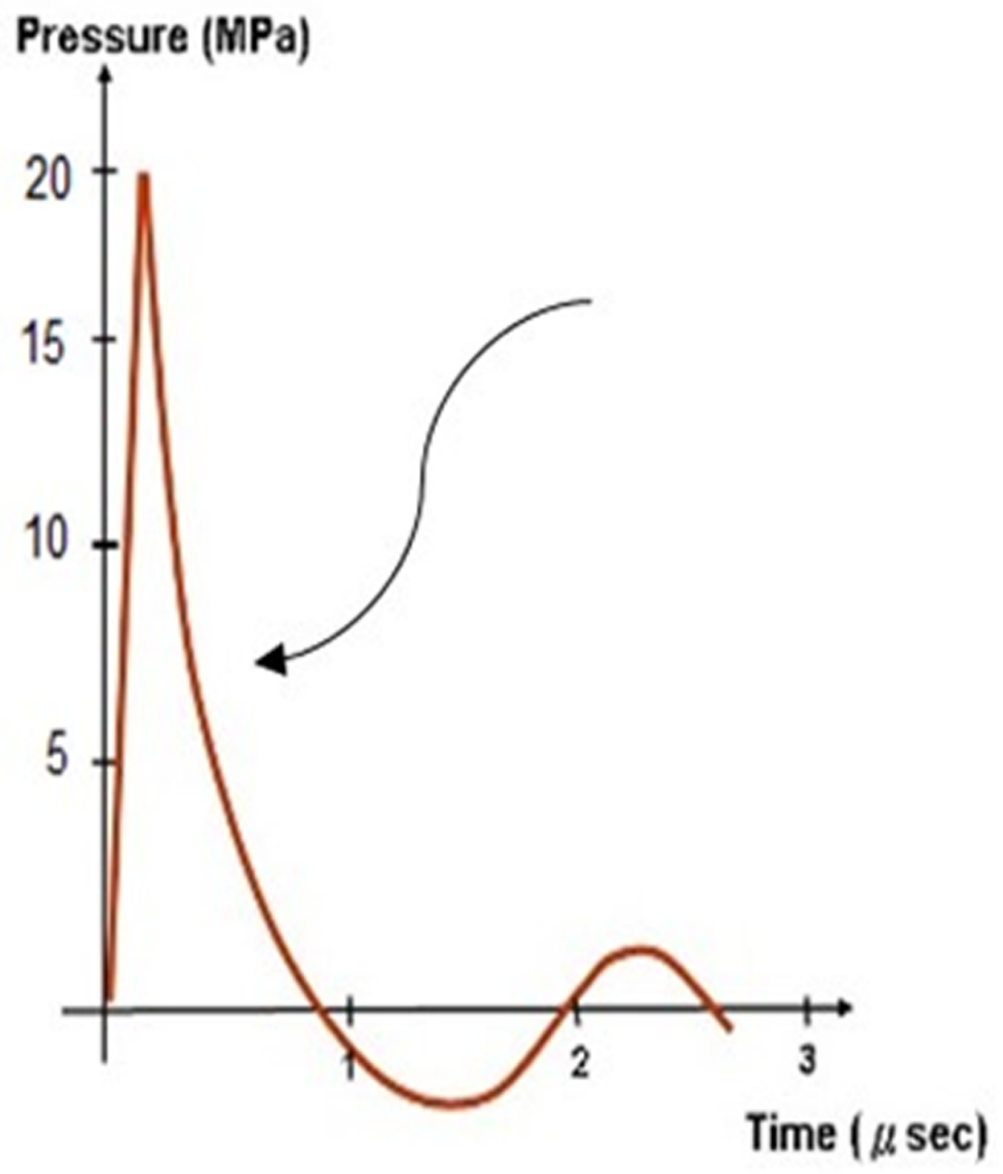
Acoustic pulse. A typical acoustic pulse in the treated area.

Acoustic pressure pulse is an acoustic wave, also called a shockwave (or radial waves), which is adapted to carry energy to body tissues (Fig 1). An acoustic pressure pulse is characterized by a major change in pressure, high amplitude and non-periodicity. The acoustic wave can be generated by kinetic energy as a result of a ballistic collision between two masses where the created energy can generate a shockwave that may be directed to biological tissue illicit a tissue treatment. The treatment capabilities of acoustic pressure pulses have been widely reported and is known to produce various responses in biological tissues, such as angiogenesis and anti-inflammatory effects [18], endorses the healing process in musculoskeletal diseases in humans [19], race horses [20] and dogs [21]. In humans, acoustic pressure pulses are also used to treat orthopedic inflammatory disorders [22, 23], heart ischemia treatment [24, 25] erectile dysfunction treatment [26, 27]. In the early 1980's acoustic pulse therapy (APT) was initially used for kidney stone fragmentation by utilizing high powered focused acoustic waves at 50–100 [MPa]. At the start of the 1990's practitioners started using ATP for the treatment of inflammations and calcification disorders (musculoskeletal diseases; [19]). Other uses of APT included use of the pressure pulse at a lower power setting of 25–35 (Mpa) [18, 22, 23]. Such a low power APT was used for orthopedic and physical therapy purposes such as: plantar fasciitis (foot pain), lateral epicondylitis (tennis elbow), medial epicondylitis (golfer elbow) and more. In the first decade of the 2000's angiogenesis effects of APT was discovered in humans.

The treatment was used for conditions such as heart ischemia, with improvement in its severity from level 4 to level 1–2 [24, 25] and as an erectile dysfunction treatment [26, 27]. The low power acoustic pulse (10–15 Mpa) was shown to produce new blood vessels and improve tissue function with long term effects. In Race horses, low power APT improved lameness disorders such as insertional desmopathies, tendinopathy, osteoarthritis myopathies, arthrosis and podotrochlosis syndrome [19, 20]. In dogs, APT was used for shoulder lameness, degenerative joint disease (DJD of the hip, elbow, knee, wrist, and ankle), Legg Calve Perthes disease (degeneration of the head of the femur bone in the hind leg) osteoarthritis and spondylosis (degeneration of joints in the spine) [21].

**Fig 2:**
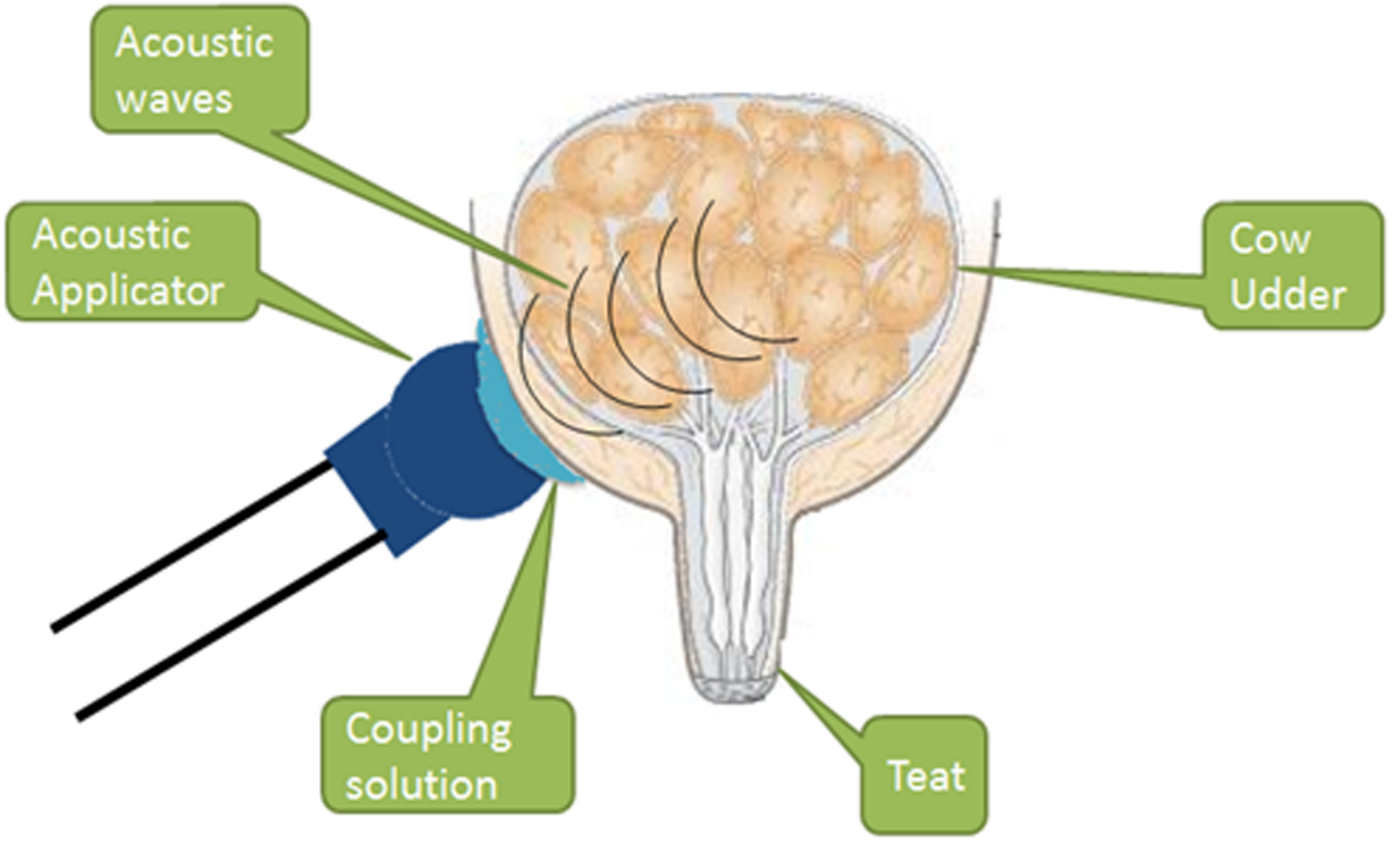
Schematic action of the acoustic pulse. A scheme of the new technological device for cow treatments producing high powered acoustic pulses distributed on a large treatment area with deep penetration via the applicator.

A new APT technology device has been developed for dairy cows. The device produces low power acoustic pulses with capability of deep tissue penetration that allows the pressure wave to be distributed over a large treatment area of the cow’s udder capable of producing therapeutic effect (Fig 2). The device’s treatment applicator is placed over the skin of to the treated gland. A special gel is used to ensure good transfer of the acoustic pulse to the treated tissue. The effect of the APT treatment produces pressure on fluid or soft tissue sites, where the generated effect is based on differences in acoustic impedance of the interface of different tissue types, for example the muscle-tissue interface.

The aim of the present study was to assess the potential of the APT technology to cure clinical and subclinical mastitis in dairy cows by using the specifically designed apparatus.

## Materials and methods

### Study layout

The study was carried out on 3 commercial dairy herds of 500–1000 lactating Israeli Holstein cows. The dairy parlors were equipped with an on-line computerized AfiFarm Herd Management system (Afimilk, Afikim, Israel) (http://www.afimilk.com) or SCR Dairy Cow Monitoring and Herd Management systems (http://www.scrdairy.com). The cows were milked thrice daily and the average milk yield (MY) was ~12,500 L during 305 days of lactation. During the study period, the monthly average bulk tank SCC varied between 170×10^3^ to 230×10^3^ cells/mL (logSCC 5.2–5.4). Routine monthly milk yield and SCC were recorded by the Israeli Cattle Breeders Association. Two parallel studies were conducted to measure the treatment effects on subclinical and clinical mastitis.

### APT treatment protocol

The APT treatment session included a total of 400 pulses (3.5 min/treatment), shockwave frequency of about 1.9Hz, at a shockwave energy density of 0.041 mJ/mm^2^. The shockwaves were delivered over two regions of the mastitis infected quarter.

On treatment days, the cows receiving the treatment were separated and brought to the parlor or restrained in the pen, where the mastitic gland was treated. The APT session took place between the first and second daily milking. Observation of cow behavior was undertaken during the APT session and post treatment until the subsequent milking. Glands were scanned for clinical reactions (redness, swelling, pain) at each milking by the milking team. Milk volume and constituents were measured in real time at each milking session by the automated milking system installed at the milking parlor.

All treatment protocols were approved by the Institutional Animal Care Committee of the Agricultural Research Organization, the government-sanctioned body for such authorizations in Israel (IL 692/17).

### 1. Subclinical mastitis

Following the routine monthly SCC a list of all cows with >6.0 cells/mL was formed. Information of lactation number, days in milk (DIM), days in pregnancy (DIP), daily milk yield (MY/d) and the SCC of the 2 previous monthly records was added from the computerized herd data. The previous SCC records helped in estimating the length of the infection with focus on the cows with first elevation of SCC. This cutoff level assured that the inflammation is active, and it focused on first elevation of SCC that suggest a new infection.

During milking, suspect quarters were examined by California mastitis test (CMT). Milk samples from cows with CMT >3 were taken for bacteriology test, SCC counts and gross milk composition analysis. Gross milk composition analysis including fat, protein and lactose content was performed with the Milkoscan FT+. SCC counts were performed with Fossomatic FC (Foss Electric, Hilleröd, Denmark). All tests were performed by the Israeli Cattle Breeders Association laboratory (ICBA, Caesarea, Israel).

Bacteriological examination was conducted according to accepted microbiological procedures of the US National Mastitis Council [28] at Kimron Veterinary Institute (Bet Dagan, Israel). Ten μL of each milk sample were inoculated onto blood agar (enriched with 5% of washed sheep red blood cells) and MacConkey agar plates (Bacto-Agar, Difco Laboratory, Becton Dickinson, Le Pont de Claix, France). Plates were incubated at 37°C and examined for bacterial growth after 18 h and 42 h. Colonies suspected to be staphylococci were tested for coagulase (tube test, Anilab, Rehovot, Israel).

Subclinical mastitis was defined when a cow’s gland had SCC >6.0 cells/mL with and/or with no bacteria finding (NBF). According to the bacteriology results, quarter milk information and cow data, each cow was grouped with 3 similar cows. Each of the 3 cows was further divided into two groups, where 2 cows were assigned to a treatment group and 1 cow for the control group. Cows were treated within a week after bacteriological testing. Cows in the treatment group were treated using the APT device (Armenta, Hod-Hasharon, Israel) 4 times, 2–3 days apart. Control group cows were not treated. All cows were retested as described above, from 3 weeks and roughly up to 2 months after the end of the treatments. Data was taken from individual cows according to the routine monthly records up to 3 months.

### 2. Clinical mastitis

Clinical udder infections were detected in one of the three herds and were diagnosed by the dairy staff and/or by the on-line computerized management system. A clinical case was defined by an inflamed gland, decreased milk yield and increased conductivity. Infected cows were segregated from the herd and moved to a separate sick cow’s yard. The herd’s veterinarian determined the course of treatment to be taken in either a control group that received antibiotics and/or NSAID or a treatment group, that was treated with the APT device according to the treatment protocol.

The clinical mastitis group included 29 cows of which the control group included 16 cows and the treatment group 13 cows. Samples from the infected quarters were taken for bacteriology analysis at time of diagnosis and again after 3 days. The clinically infected cows were treated twice with APT using the same protocol. In order to follow up the clinical infected cows, the infected quarters were tested 2 more times, 2 and 4 weeks thereafter for bacteria presence and milk composition, including SCC. Cow performance was taken from the routine monthly’s record up to 100 d.

### Statistical analysis

Two different models were produced. The first analysis was carried out using the mixed procedure of SAS [29], with the general form: Result = treat + herd + parity + DIM + error. Where: treat = two groups of dairy cows, the first one was treated and the second served as control group, herd = three different dairy farms, parity = 1^st^, 2^nd^, or 3 and more lactations, DIM = days in milk at time of treatment.

The second analysis used the General Linear Model procedure of SAS [29], with the general form: Dependent variable = result + treat + parity + herd + error. Where: result = success or failure of the treatment given, treat = two groups of dairy cows, parity = 1^st^, 2^nd^, or 3 and more lactations, herd = three different dairy farms. The dependent variables were: logSCC prior to treatment, logSCC after treatment, milk levels prior to treatment, milk levels after treatment, lactose prior to treatment and lactose after treatment. Data are presented as means and SEM.

Recovery criteria from the infection was defined as cow milk logSCC <5.6 cells/mL monthly during the first 100 days following treatment and milk of the treated quarter appear normal: logSCC <5.6 cells/mL and lactose > 4.8%.

The effects of bacteria cure were compared separately for *Escherichia coli*, *Streptococci* and CNS by means of the chi-squared (χ^2^) test. At time of treatment, DIM had no effect on treatment result and were therefore excluded from the second model that tested parameters before and after treatment.

## Results

According to routine monthly milk records, 116 cows with high LogSCC (>6.0 cells/mL) were enrolled to the study along with 29 cows from 1 herd that were diagnosed with clinical mastitis by the herd’s veterinarians.

No adverse reactions were observed during the APT sessions, except for slight signs of nervousness at the beginning of every treatment session in reaction to touching during the application of gel on the treated quarter. No inflammatory reaction was noted on the day of treatment or the day after and milk production remained stable.

### 1. Subclinical mastitis

Overall, in the 3 herds, 116 cows were identified with 1 inflamed quarter per cow with or without identification of the causative bacterium and were assigned to treatment (n = 78) or control (n = 38). The treatment group was composed of 21 cows with NBF and 57 cows with one of 4 identified bacteria, *E*. *coli*, *Streptococci*, CNS or *S*. *aureus*. The control group was composed of 6 cows with NBF and 32 cows infected with similar bacteria (Table 1).

**Table 1:** Distribution of 116 cows from 3 dairy herds according to treatment group, herd and bacteria specie.

Group	Herd	Cow	Bacterium				
			*E*. *coli*	Strep.	CNS	*S*. *aureus*	NBF
Treatment	1	32	3	7	14	-	8
	2	25	2	10	5	-	8
	3	21	-	5	7	4	5
	Total	78	5 (6.4%)	22 (28.3%)	26 (33.3%)	4 (5.1%)	21 (26.9%)
Control	1	18	-	5	11	-	2
	2	15	5	5	3	-	2
	3	5	1	1	1	-	2
	Total	38	6 (15.8%)	11 (28.9%)	15 (39.5%)		6 (15.8%)

Strep.—Streptococci

CNS—coagulase negative staphylococci

NBF—no bacterial finding

**Fig 3:**
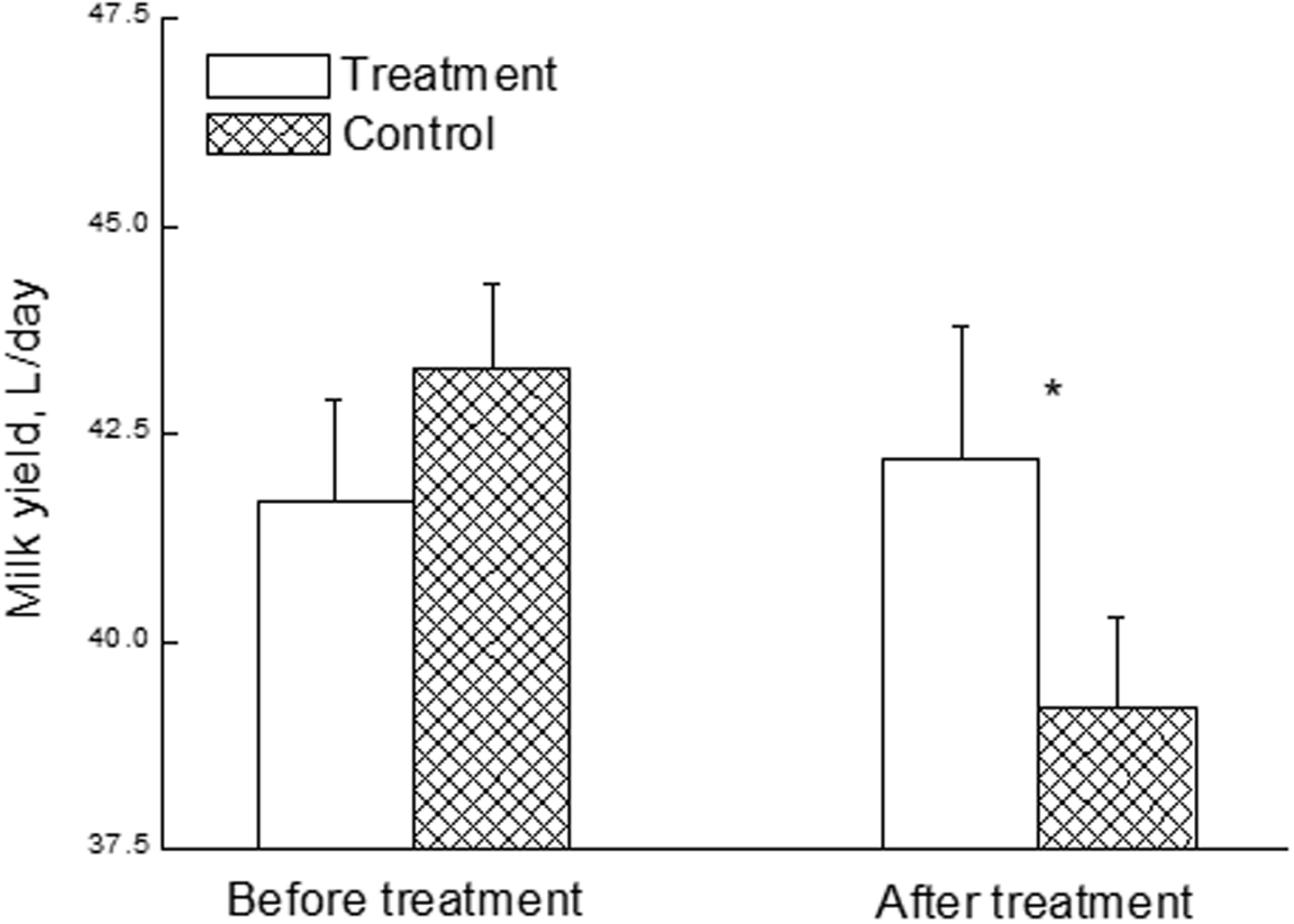
Daily milk yield. Daily milk yield of 116 cows from 3 commercial herds identified with subclinical mastitis, before treatment and up to 3 months after it according to APT treatment (n = 78) and control (n = 38).

No interaction was found with herd, lactation, DIM, MY/d. Some differences were found related to the bacterium specie, of which none were significant, and therefore further analysis was conducted on these parameters. Of the treated cows, 70.5% responded successfully to the treatment, while in the control group only 18.4% returned to producing normal milk (Table 2). In comparison between treatment and control, MY/d of the treated cows increased significantly (*P<0*.*05*) compared to the quantity prior to treatment and subsequently (from 41.7 to 43.3 L/day), compared to decreased MY/d of the control cows (from 42.2 to 39.2 L/d) (Table 2 and Fig 3).

**Table 2:** Log somatic cell count (logSCC) and milk yield on the cow level and % lactose on the quarter level, before treatment and up to 3 months according to result—Success or failure, of 116 cows from 3 dairy herds treated or not with acoustic pulse therapy (APT). P[*F*] treatment vs. control.

Group	Result	Cow, n (%)	logSCC		Milk yield (kg/d)		Lactose (%)	
			pre	post	pre	post	pre	post
Treatment	Success	55 (70.5)	6.42±0.04^a^	5.23±0.12^b B^	40.2±1.5	43.2±1.1	4.84±0.06 ^b^	4.94±0.08 ^a A^
	Failure	23 (29.5)	6.32±0.04	6.23±0.10 ^A^	45.4±1.9	43.6±2.4	4.57±0.13 ^a^	4.22±0.15 ^b B^
	Total	78	6.39±0.03	5.52±0.11	41.7±1.2	43.3±1.0	4.76±0.06	4.75±0.08
Control	Success	7 (18.4)	6.4.5±0.12^a^	5.49±0.10^b^	43.4±2.7	44.5±2.8	4.52±0.32 ^b^	4.90±0.15 ^a A^
	Failure	31 (81.6)	6.32±0.05	6.00±0.0.8	41.9±1.9	37.5±1.7	4.34±0.17	4.31±0.20 ^B^
	Total	38	6.35±0.05	5.89±0.08	42.2±1. 6	39.2±1.1	4.44±0.17	4.55±0.16
P [*F*]			NS	< 0.001	NS	< 0.05	NS	NS

Results are presented as mean ± SE;

Parameters within rows (a,b) and between columns (A,B) with no common letters differ significantly (*P < 0*.*05*).

**Fig 4:**
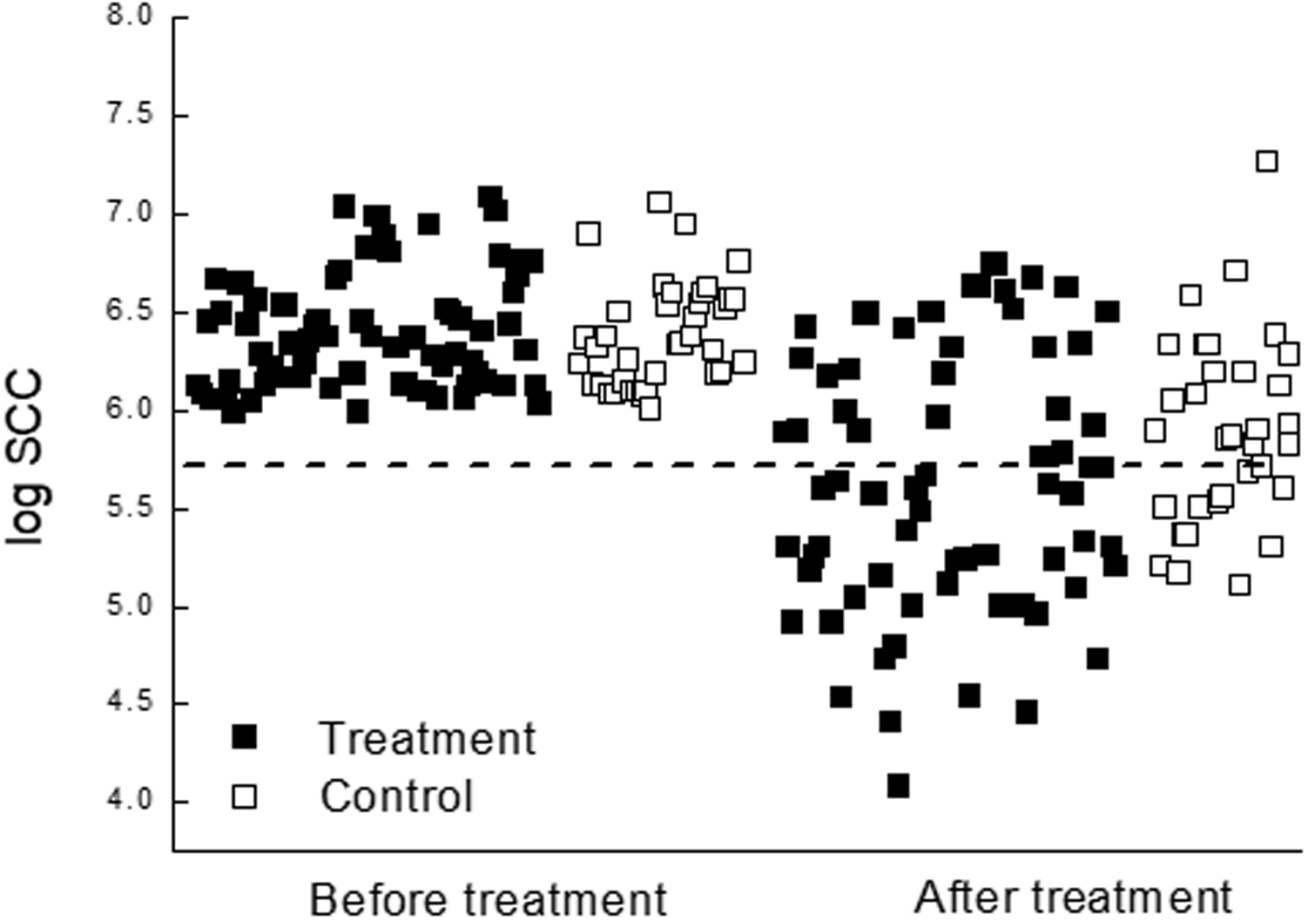
Distribution of somatic cell count. Distribution of individual logSCC of 116 cows from 3 commercial herds identified with subclinical mastitis, before treatment and up to 3 months after it, according to Acoustic pulse therapy (APT) treatment (■; n = 78) and control (□; n = 38).

SCC count before the study did not show significant differences between the subclinical groups (Table 2 and Fig 4). The percentage of the treated cows with logSCC under 5.6 cells/mL and the milk of the infected quarters that appeared normal with lactose >4.8% were significantly higher (*P<0*.*001*) than of the control (Table 2).

Of the treated cows with identified bacteria (57 cows), 52.6% of the quarters were cured from the infection, while in the control only 25.0% were cured (Table 3). The difference between the spontaneous cure (of the control animals) and cure of the treated cows was highly significant (*P*<0.001). Individual data of all cows/quarters are presented in S1 Data.

**Table 3:** Bacteria cure of 116 cows from 3 dairy herds treated or not with acoustic pulse therapy (APT). P[*F*] treatment vs. control.

Bacteriology	Treatment			Control			P [*F*]
	pre	post	% Success	pre	post	% Success	
*E*. *coli*	5	0	100.0	6	2	66.6	<0.001
*Streptococci*	22	14	36.4	11	9	18.2	<0.001
CNS	26	12	53.8	15	13	13.3	<0.001
*S*. *aureus*	4	1	75.0	-	-		
Total bacteria positive	57	27	52.6	32	24	25.0	<0.001
NBF	21	No new infection		6	No new infection		

CNS—coagulase negative staphylococci

NBF—no bacterial finding

Among the bacteria, all cows identified with *E*. *coli* and treated were cured compared with 66.6% spontaneous cure in the control as shown in Table 3. Spontaneous cure of the CNS and *Streptococci* was low while the treatment successfully increased the cure of CNS from 13.3% in the control to 53.8% in the APT group and *Streptococci* from 18.2% in the control group to 36.4% in APT group. Of the 4 cows identified with *S*. *aureus*, 3 were cured.

### 2. Clinical mastitis

Overall, 29 cows were identified with clinical infection and were submitted either to antibiotic/NSAID treatment (n = 16) or APT treatment (n = 13). In both groups the cause of infection was *E*. *coli*, *Streptococci* or with NBF (Table 4). Overall, of the antibiotic/NSAID treated cows 18.7% were cured from the bacteria and the SCC count was reduced to <5.6 cells/mL. Moreover, 56.3% of these cows which did not recover, were culled (9/16). In comparison, the APT treatment resulted in a significant cure (P <0.001) and 76.9% of the cows returned to the herd, while only 1 cow was culled (7.7%). As can be seen in Table 4 of the different bacterial infection groups the lowest success was observed with cows infected with *Streptococci*, however this may be attributed to the low sample size in this sub-group.

**Table 4:** Bacteria cure and cow somatic cell count (SCC) of 29 cows treated with antibiotic (16) or with acoustic pulse therapy (APT) (13).

Treatment	Time	Bacteriology									
		*E*. *coli*		*Streptococci*		CNS		NBF		Total	
		Bac	logSCC	Bac	logSCC	Bac	logSCC	Bac	logSCC	Bac	logSCC
Antibiotic	Pre	3	>6.70	5	>6.70			8	>6.70	16	>6.70
	Post	2	6.11	2	6.53			2	5.33	6	6.28
	Cull	1		2				6		9	
	Success^1^ (%)	0/3 (0.0)		1/5 (20.0)				2/8 (25.0)		3/16 (18.7)	
APT	Pre	8	>6.70	3	>6.70	1	>6.70	1	>6.70	13	>6.70
	Post	1	5.49	2	6.23	0	5.37	0	5.47	3	5.87
	Cull	1								1	
	Success^1^ (%)	7/8 (87.5)		1/3 (33.3)		1/1 (100.0)		1/1 (100.0)		10/13 (76.9)	
P [*F*]		<0.001		NS				<0.001		<0.001	

^1^ Success = cure of bacteria and logSCC <5.6 cell/mL after 20–50 days

Bac—bacteriology

CNS—coagulase negative staphylococci

NBF—no bacterial finding

## Discussion

Despite many years of efforts to control mastitis, this infection is still one of the leading causes of economic losses to the dairy industry worldwide. When estimating the loss due to mastitis, it is important to consider which factors are economically important [30, 31], and whether the estimates include clinical mastitis alone or include subclinical mastitis as well. Estimating losses of clinical mastitis are straightforward: the infection is visible and requires treatment due to animal welfare. Dealing with subclinical mastitis presents different challenges because in many of the cows there are no recognizable symptoms and the milk appears normal. If the bulk tank milk is not paid a reduced price due to lower quality, those cows are ignored. Calculations of subclinical mastitis include various costs, such as: diagnostics of the infecting agent, veterinary services, medication, labor, discarded milk, decrease in milk production, premature culling and mortality.

In exploring alternative treatments, some of the above factors do not change while others do. When antibiotic is used, diagnostics of the causative bacteria and susceptibility test are suggested before treatment. However, one of the major losses from antibiotic treatment is the milk discarded during and after treatment due to antibiotic residues. This non-saleable milk can rise to above 50% of the mastitis treatment costs. Moreover, this single factor is the major cause for not treating subclinical mastitis during lactation with the distressing concern of overuse of drugs [32]. Therefore, non-antibiotic alternative treatments in farm animals are highly desirable. Treatment requires: 1. Identification of infection, where timing from bacterial invasion is critical; 2. A high successful cure of the bacterial infection and limiting the destruction of the gland by the immune system are desirable. Intramammary infections with bacteria such as *E*. *coli* can lead to long-lasting negative effects on the quantity and quality of the gland’s milk, which persist for months even after the bacteria are eradicated [33]; 3. Reasonable cost.

The major advantage of the new APT technological device over antibiotics are derived from making diagnostics of the bacteria, including susceptibility test—needless, reduced veterinary services, lesser use of medications and most important, a significant decrease in non-saleable milk owing to antibiotic residues. The treatment mechanisms of antibiotics and APT are different. Drugs are directed to kill and/or to slow down growth of bacteria, thus allowing the immune cells to eradicate the bacteria but not in helping to regenerate the damaged tissue. The ATP treatment is directed to increase angiogenesis and anti-inflammatory responses in the mammary tissues. Therefore, not only increase the activation of the immune cells but also accelerate the recovery of the regenerated gland tissue.

Acoustic pulse therapy–APT, opens an opportunity for treating subclinical mastitis during lactation, increasing the possibility of higher MY and quality and may lead to reducing the need for dry therapy. In the present study, only 29 cows with clinical mastitis were treated with antibiotics or APT, regardless of the bacteria. The results revealed a higher cure of the bacteria, a faster return to milking into the bulk milk tank and to the level of SCC as prior to the infection incident, as well as lower culling than that of treating with antibiotics.

Nevertheless, the major benefit was demonstrated by treating subclinical mastitis during lactation. Because no negative effects on MY and other milk parameters were observed, cows were milked normally during treatment, so no loss of milk accrued. Only 18.4% of the control cows that were identified with subclinical infection but were not treated, returned to producing normal milk. However, only 60% of *E*. *coli* infections, 18.2% of *Streptococci* and 13.3% of CNS were cured without intervention. In contrast, when applying the APT treatment, 70.5% of the cows were cured and returned to producing normal milk with a significant increase in MY. Moreover, quarters of treated cows with identified bacteria had a bacterial cure of 52.6%. Among the bacteria, all cows which were identified with *E*. *coli* and treated were cured, as well as cows identified with *Streptococci* (36.4%) or with CNS (53.8%) and *S*. *aureus* (75%). These results are highly important due to the high cost of the alternative treatment (antibiotic), which is in fact not conducted. The new APT treatment of clinical and subclinical mastitis can: 1. significantly reduce the use of antibiotics; 2. significantly reduce milk discarding during treatment; 3. can be used to treat subclinical mastitis during lactation; 4. improve milk quantity and quality during the lactation, probably due to the increased healing process of the damaged tissues; 5. decrease culling of subclinical mastitis cows due to low milk production and low milk quality.

Therefore, cow longevity in the cowshed can be increased and forced culling can be reduced. Thus, the percent of replacement heifers will be reduced, and milk production will be increased, as older cows produce more milk than young ones (+15%). Moreover, this can lead to holding fewer cows for producing the same quantity of milk, saving food, lowering treatment expenses and labor and obtaining higher milk production. Repeated treatments could also increase recovery success for SCC and microbial improvement.

When fully adopted, the annual economic potential of the APT is $15 million in Israel (~112,000 cows) and expanding it to the USA and Europe (~20 million cows), it may reach $ 1 billion. This analysis is based on assumptions in terms of management strategy and economic conditions, but it suggests that the technology has a very promising economical future.

## Conclusions

Acoustic pulse therapy—APT—is more effective than antibiotics or no-intervention in treating clinical and subclinical mastitis in dairy cows. In contrast to current treatment options for subclinical mastitis, which require early detection, APT is an easy to use confined treatment of cow’s udders. It does not require bacterial identification nor discarding of milk during and after treatment. Consequently, it is suggested that every cow suspected to be affected with any form of mastitis should be treated with the APT apparatus to gain back loss of milk production.

## Supporting information

S1 Data Individual cow data. (XLSX)
